# Nutritional Peak Week and Competition Day Strategies of Competitive Natural Bodybuilders

**DOI:** 10.3390/sports6040126

**Published:** 2018-10-24

**Authors:** Andrew J. Chappell, Trevor N. Simper

**Affiliations:** Food and Nutrition Group, Sheffield School of Business, Sheffield Hallam University, Howard St., Sheffield S1 1WB, UK; t.simper@shu.ac.uk

**Keywords:** bodybuilding, drug free, competing, peaking, carbohydrate loading, water loading, sodium loading, fat loading, Vitamin C, fibre restriction

## Abstract

Bodybuilders utilize peaking strategies in a bid to fine-tune their aesthetics for competition day. The most prevalent peaking strategies utilized by natural bodybuilders are unreported in the current literature. Eighty-one (M-59, F-22) natural bodybuilders were recruited from competitions during the 2016 and 2017 British Natural Bodybuilder Federation seasons. Competitors completed a 34-item questionnaire designed to investigate peaking and contest day strategies. The questionnaire listed commonly utilized peaking strategies and provided additional space for qualitative information. Analysis of the data indicated that carbohydrate (CHO), water, and sodium manipulation were the most commonly utilized peaking strategies. The consumption of high glycemic index CHO was the most common competition day strategy. Only 6.2% of competitors reported following their regular diet the week prior to competition. The CHO manipulation strategies followed were similar to classical CHO loading, whereby bodybuilders attempt to maximize muscle glycogen concentrations. Furthermore, bodybuilders attempted to remove superfluous water by exploiting the diuretic/polyuria effect associated with water loading/restriction. The potentially deleterious effects of peaking on bodybuilders’ health is considered and the efficacy of these strategies to enhance appearance is discussed. The findings of the present investigation are likely to be of interest to bodybuilders and their coaches.

## 1. Introduction

In competitive bodybuilding, athletes are judged on muscle size, conditioning (appearance of low body fat), and symmetry (muscular proportions) [1]. To obtain the desired physique, bodybuilders employ strict dietary and training regimes, in the months prior to competition [2,3,4,5]. In the week prior to competition, bodybuilders also employ tapering strategies for “fine-tuning the body” in an attempt to maximize their contest day aesthetics [6,7,8]. Known as “peaking” or “peak week”, these strategies involve the manipulation of macronutrients, electrolytes, water, and exercise [6,8,9]. The main goals of peaking are: (1) To increase “muscle fullness”, by maximizing muscle glycogen content; (2) to obtain a “dry” or “hard” look, by minimizing subcutaneous water; and, (3) finally, maximizing the “V-taper”, by minimizing abdominal bloating [6,8,10,11,12].

There is a lack of scientific literature published on the peaking strategies of competitive bodybuilders. Only a single trial to date has investigated the effects of carbohydrate loading (CHOL) on muscle girth, finding no effect [9]. This study replicated the popular “Aceto/Addison” peaking method, however, it was performed on a non-bodybuilding population under isocalorfic conditions [9,11]. Despite these findings, CHOL is popular amongst bodybuilding populations [8]. Peaking for success, however, is known to be challenging and stressful, while poorly conceived strategies can be detrimental to performance [8]. Peaking strategies are often self-prescribed or designed by coaches, the safety of which has been called into question [13]. This point is emphasized by the fact a recent study reported that only 14.1% of bodybuilding coaches were qualified nutritionists/dietitians [1,14]. Moreover, a qualitative study of bodybuilders reported that athletes felt there was a lack of scientific nutritional knowledge amongst coaches [8]. Therefore, observing the peaking strategies used by competitive bodybuilders, as well as discussing their potential mechanisms of action would be of value to the bodybuilding community. This cross-sectional investigation aims to detail and describe peaking strategies, and is likely to be of interest to bodybuilders and coaches seeking to improve their understanding of the pre-competition phase.

## 2. Materials and Methods

### 2.1. Experimental Approach to the Problem

Male and female competitors participating in the British Natural Bodybuilding Federation (BNBF) championship qualifiers in 2017 submitted data, which was then combined with a previous dataset from the 2016 BNBF British championship [3]. All competitors were subject to the same drug testing and polygraphing criteria explained previously [3]. Drug testing was carried out on all class winners at regional qualifiers, alongside targeted testing in accordance with the World Anti-Doping Agency prohibited list [15,16]. Recruitment was performed by the first author (AC) as described previously [3]. All participants were informed of the study aims and methods via a participant information sheet, and those agreeing to take part provided written informed consent. Each participant then completed a 34-item questionnaire (see Supplementary Material S1), that inquired about dietary and training habits, weight change, and peak week and competition day strategies. The questionnaire provided a list of commonly utilised peaking strategies, as well as space for participants to provide additional qualitative information on those strategies. Qualitative quotes were counted and grouped based on the peaking strategies they related to and representative quotes are provided for context. Participants provided varying amounts of qualitative data; some competitors provided detailed accounts of strategies utilised, while others provided short statements. Qualitative quotes are presented verbatim. Missing questionnaire data and clarification of strategies were followed up via email. The most commonly followed peaking strategies were counted, and are presented as a percentage of the total population. This investigation was approved by the Sheffield Hallam University School of Business Ethics Committee. Ethics application number SBS-191, approved 19 September 2016.

### 2.2. Participants

Eighty-two participants were recruited for the present investigation. One competitor was excluded after failing a pre-competition polygraph test. The final data set included 81 competitive natural bodybuilders (*n* = 59 male, *n* = 22 female). Participant characteristics are detailed in Table 1. Competitors provided their self-reported weight prior to starting their contest diet and their weight the day prior to competition. Total weight loss, the difference in weight loss, and body mass index (BMI) (kg/m^2^) was calculated with self-reported height. The male data set was comprised entirely of bodybuilders from the following classes: Teens (*n* = 4), under 23 years (*n* = 8), novices (*n* = 10), open weight (*n* = 20), masters (*n* = 13), and professional (*n* = 5). All female competitors were also grouped together and were recruited from the following classes: Figure (*n* = 15) open (*n* = 9), over 40 years (*n* = 4), professional figure (*n* = 2), athletic (*n* = 5), and bodybuilding (*n* = 3). It is worth noting that figure and athletic classes place less emphasis on muscle size compared to bodybuilding, body fat levels are distinctly different between athletic (lower) and figure (where it is higher).

## 3. Results

### 3.1. Peak Week Strategies

The strategies utilised during peak week are detailed in Table 2. Of the 81 competitors surveyed, only 5 (6.2%) reported following their ‘regular diet’ in the week prior to competition (i.e., they did not employ a specific peaking strategy). Peaking strategies were not always mutually exclusive, and competitors employed multiple strategies sometimes simultaneously e.g., CHO restriction combined with loading.

**Figure 1:**
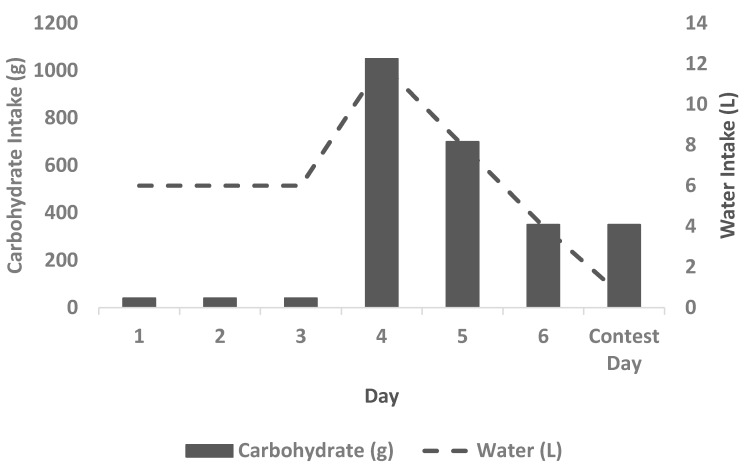
The Aceto/addision peak week. A proposed peaking plan based on an 80 kg bodybuilder with a daily carbohydrate (CHO) intake of 350 g per day (4.4 g/kg BW). Three days of CHO restriction (40 g per day, days 1 to 3) is employed, followed by two days of loading. High intensity aerobics and high volume resistance training is employed during the CHO restriction phase to deplete muscle glycogen stores. During the CHO loading phase, the regular dietary CHO intake is multiplied by three (1050 g, 13.2 g/kg BW) and two (750 g, 9.4 g/kg BW) on days 4 and 5, respectively. Intake on day 6 (day prior to competition) returns to the regular dietary intake (350 g (4.4 g/kg BW) and coincides with water restriction. The total CHO intake over this three day period is 2150 g. The majority of CHO consumed on competition day are consumed pre-stage. Water intake parallels CHO intake, peaking on day 4, before water restriction is imposed 12 to 16 h prior to competing. Water may be completely restricted or reduced to sipping on contest day. Finally, sodium manipulation and vitamin C loading may be introduced on days 5, 6, and contest day. Abbreviations: CHO = carbohydrate, BW = bodyweight.

Carbohydrate manipulation was the most common peaking strategy; qualitative quotes indicated that restriction and loading lasted between one and four days, with restriction preceding loading (Table 3). Carbohydrate intake during the restriction phase varied and competitors reported consuming between 0 and 100 g per day. Conversely, CHO intake during loading was reported to be over 2500 g or 833 g per day (11.1 g/kg bodyweight (BW) in a 75 kg bodybuilder) amongst three male competitors. Bodybuilders reported consuming white and sweet potatoes, oats, confectionary, white rice, grapes, and bananas during CHOL. Water manipulation was the most popular strategy after CHO manipulation. The amount of water consumed during the loading phase varied between 4 to 12 L per day (53.3 to 160 mL/kg BW in a 75 kg bodybuilder) (Table 3). Water loading preceded restriction, with competitors reducing their water intake as they approached the competition. Ten to 24 h prior to competition, competitors reported employing water restriction strategies. Competitors also loaded and restricted sodium in the days prior to competition. Qualitative quotes indicated that sodium manipulation was practiced three to four days prior to competition (Table 3). Quotes indicated that there was no consistent order for sodium loading/restriction strategies, i.e., some competitors restricted prior to loading and others vice versa. Finally, competitors reported megadosing with vitamin C (VITC) (1 to 8 g per day) in the days preceding competition. Other strategies employed included protein and fat loading as well as the use of dandelion tea. A graphical representation of a common peaking plan is provided in Figure 1 for reference purposes.

### 3.2. Competition Day Strategies

Twenty-one male (35.6%) and 4 female (18.2%) competitors reported following their regular diet on competition day, although many of these competitors employed one specific additional strategy of extra CHO intake pre-stage. Details of competition day strategies and commonly consumed contest day foods are provided in Table 4, Table 5 and Table 6.

The consumption of high glycaemic index (GI) CHO prior to stepping on stage was the most widely used contest day strategy. Fruit, confectionary, and preserves were the competitors preferred choice of CHO during the pre-stage period (Table 5). A high CHO intake persisted from peak week and competitors reported consuming rice cakes, white and sweet potatoes, oats, and rice on competition day (Table 5). Water restriction also continued from peak weak, with competitors reporting minimal or restricted intake on contest day (Table 6). A low fibre diet via the exclusion of fibrous vegetables was the most common strategy after CHO and water manipulation. Alcohol and sodium loading prior to competing was also reported and competitors opted for spirits or wines and salty snacks, and/or adding salt to meals. High protein and fat strategies involved competitors grazing on foods high in protein and fat or where competitors opted for high protein and fat breakfasts (Table 6). Other contest day strategies included water loading, the consumption of B-vitamin supplements, the use of arginine based supplements, and the restriction of both CHO and food to reduce bloating.

## 4. Discussion

This study aimed to identify peaking strategies utilised by competitive natural bodybuilders. Analysis of the data indicated that 93.8% of competitors were engaged in peaking, with CHO and water manipulation being the most prevalent peak week strategies. Carbohydrate manipulation strategies were similar to the strategies described by Balon et al. [9] and Aceto [11]. Mega dosing with VITC and sodium manipulation was also utilised during peak week. Contest day nutrition focused on the consumption of high GI CHO, low fibre intake, and, in some cases, was combined with water restriction and alcohol consumption. These findings are in agreement with Mitchell et al. [8] and Alwan et al. [14] who reported a similar focus on CHO, water, and sodium manipulation during peak week amongst bodybuilders and physique competitors. These findings also reflect the deviation from the regular diet noted by Spendlove et al. [7] amongst bodybuilders in the weeks prior to competition. To our knowledge, this is the first study to attempt to quantify the prevalence of peaking strategies amongst natural bodybuilders. This investigation may, therefore, provide a useful starting point for researchers to identify which peaking strategies warrant further investigation. This study also provides additional qualitative comments on how these peaking strategies are employed.

### 4.1. Peak Week

#### 4.1.1. Carbohydrate Manipulation

Carbohydrate manipulation strategies followed a similar pattern to classic CHOL, with three days of restriction, followed by three days of CHOL [9,17]. Although competitors did not indicate if they altered their exercise routine during the restriction phase, bodybuilders are known to employ high volume resistance training during this period [8]. The addition of exercise alongside CHO restriction aims to deplete muscle glycogen, as skeletal muscle lacks glucose-6-phosphotase and therefore cannot contribute to maintaining blood glucose [18]. Furthermore, CHOL following CHO depletion may result in greater glycogen synthesis activity, enhanced glucose transport, and increased muscle glycogen supercompensation (MGS) [19]. Moreover, studies in animal models indicate greater upregulation of glycogen synthase and glucose transporter type 4 mRNA, following glycogen depletion; while depleted muscle tissue has increased insulin sensitivity over 48 h dependent on the initial glycogen content [20,21,22]. Energy intake is inevitably reduced as a consequence of CHO restriction. It may therefore be prudent for bodybuilders to increase their fibre and protein intake during this phase as a way of compensating for the loss of energy and the additional satiating effect associated with these nutrients [23].

Carbohydrate intakes of 8 to 10.5 g/kg BW per day during CHOL have been demonstrated to produce MGS [18,24]. This equates to a CHO intake 600 to 785.5 g, or 1800 to 2362.5 g over three days for a 75 kg bodybuilder. Interestingly, three competitors who quantified their CHOL regime achieved an intake greater than or equivalent to these levels. While bodybuilders may wish to take a more conservative approach to CHOL to prevent “spilling over” (too much CHO is thought to result in a watery looking physique) [11,12]; lower CHO intake may be inadequate to achieve MGS. It is worth mentioning that traditional CHOL regimes may not perfectly translate from endurance sport to bodybuilding, e.g., marathon runners are not concerned with CHOL’s effect on physical appearance. Conversely extending exercise output is not the goal of a competitive bodybuilder; rather it is full, dry looking muscles. High GI CHO was prioritised at the start of CHOL, before competitors reported moving onto lower GI sources. Carbohydrate loading strategies varied, although, “front-loading”, where most of the CHO was consumed initially, was the most prevalent. This front-loading and the initial use of high GI CHO reflect the notion that glycogen synthesis and storage may be greater in the initial hours following glycogen depletion [20,21,22]. Front-loading may also suggest a pragmatic approach as bodybuilders seek to reduce CHO intake closer to competition to reduce unnecessary gastrointestinal or psychological stress associated with peaking [8]. 

Modified, and single day CHOL combined with high intensity sprint exercise, has also been demonstrated to be effective at producing MGS [25,26]. One-day protocols could be utilised by bodybuilders and their practicality requires consideration. However, the bodybuilder’s sole goal during peak week is not MGS as multiple variables of the diet and training are manipulated simultaneously. Bodybuilders are aiming to enhance the appearance of muscle size that is thought to be achieved from MGS, while also reducing subcutaneous water, which enhances the appearance of definition. Longer peaking strategies provide bodybuilders with time to adjust their strategies depending on their day to day appearance. Furthermore, conventional bodybuilding wisdom advocates two to three days of rest prior to competition [11]. Bodybuilders close to competition may therefore perceive the high intensity sprint exercise employed alongside single day CHOL negatively, although if timed correctly, a single bout of sprint exercise is unlikely to result in delayed onset muscle soreness, or loss of isometric capacity. Single day plans would also reduce the interruption to regular training and pre-contest diet seen when weeklong peaking plans are employed. This may allow the competitor to lose more body fat during this time saved in preparation for competition. Single day plans may therefore represent a viable less stressful alternative to the classical approaches currently employed. Finally, loading strategies should consider the athlete’s prior dietary approach and low CHO, high fat diets have been demonstrated to decrease both insulin receptor and glucose transporter type 4 mRNA expression [27]. Despite these findings, CHOL when employed following acute fat adaptation still results in MGS [28], although the effect this approach has on the athlete’s physical appearance is unknown.

#### 4.1.2. Water, Electrolytes, and Vitamin C

Bodybuilders manipulate water during peak with the goal of facilitating MGS and removing superfluous subcutaneous water. Water manipulation strategies paralleled CHO manipulation, with high initial intakes followed by a gradual reduction approaching competition day. Previously, Balon et al. [9] and Reale et al. [29] noted that between 2.3 to 7.8 mL of water is stored per g/glycogen. This would equate to a requirement of 1000 to 3600 mL of water in a 75 kg bodybuilder with a CHO store of 462 g. When the bodybuilders’ habitual water requirements are considered alongside CHOL, the consumption of additional water to facilitate MGS may be merited. A number of competitors reported consuming between 8 to 12 L of water per day (106.6 to 160 mL/kg of bodyweight, 75 kg bodybuilder) during the loading phase, likely meeting their habitual and CHOL water requirements. Moreover, excessive water consumption causes polyuria, and bodybuilders seek to exploit this diuretic effect, prior to imposing water restriction with the aim of removing any superfluous water [29,30]. Bodybuilders, however, should be mindful that skeletal muscle is largely water, and dehydration may negatively affect their appearance, where muscles could potentially end up “flat” looking, lacking in volume or size [31]. Water manipulation combined with MGS may offer some protection against going “flat”, as muscle glycogen is exclusive for skeletal muscle metabolism. Water bound to glycogen would be retained intracellularly provided the bodybuilder refrained from exercise, although extracellular water may still be lost, negatively affecting appearance. Researchers should however, be mindful that peaking strategies might run counter to traditional sports nutrition practice, and bodybuilders will actively seek to dehydrate themselves to obtain a desired “look” at the expense of metrics, like aerobic or anaerobic performance. The success of peaking strategies should therefore be judged on real-world aspects, such as the bodybuilder’s competition outcome, physical appearance and performance relative to other competitors, and past performances.

Bodybuilders also attempt to remove subcutaneous water via sodium loading and restriction, while it’s worth noting that peak week diets are high in potassium rich foods, e.g., bananas, sweet, and white potatoes. Potassium and sodium are intracellular and extracellular cations, both of which maintain: cellular bioenergetics, integrity, and fluid balance via gated pumps [32]. Moreover, the renal system regulates fluid balance and osmotic pressure by excreting or retaining sodium and potassium depending on their relative concentrations [33]. Exercise scientists have long been aware that the addition of electrolytes to water is effective at enhancing hydration [34], while the depletion of these electrolytes results in a loss of fluid, and a reduction in both blood pressure and plasma volume [29]. Manipulation of these electrolytes could therefore potentially alter fluid balance and enhance a bodybuilder’s appearance. It was not clear in the present investigation if sodium loading or restricting was the preferred method amongst competitors, although, the “Aceto” peaking method recommends restricting sodium three to four days prior to competition [11]. It is worth noting though that sodium is required as a cotransporter for the uptake of glucose within the small intestine via sodium glucose linked transporter 1 [35]. Restriction of sodium three to four days prior to competition may therefore affect the efficacy of CHOL and the subsequent MGS. A lack of consensus amongst competitors on electrolyte manipulation may also reflect the complexity of adding this additional variable to peaking plans, the outcome of which may be difficult to predict alongside CHO and water manipulation.

Finally, bodybuilders reported mega dosing with VITC three to four days prior to competition during the water-loading phase. High VITC consumption is known to stimulate diuresis and bodybuilders use VITC in an attempt to remove excess (subcutaneous) water [36,37]. Mega dosing with VITC may cause gastrointestinal issues, while chronic dosing may cause acidification of the urine, and increasing the risk of urate renal stones [38]. Ascorbic acid and urine excretion increases significantly with dosages of VITC over 200 mg [39]; it is therefore plausible that bodybuilders may be able to obtain the same diuretic effect with lower dosages than those reported in the current investigation [39]. Furthermore, employing the diuretic effect of VITC during CHOL and water loading may reduce the efficacy of MGS. Vitamin C loading may therefore be better utilised once the initial CHOL regime is complete to remove subcutaneous water. The consumption of high amounts of herbal tea and protein were other strategies utilised by competitors in an attempt to remove excess water. Such strategies may have some merit as high protein diets are known to increase urea production and glomerular filtration rate both acutely and chronically [40].

### 4.2. Competition Day Strategies

Although competitors reported returning to their regular diet for the competition day following peak week, many of these bodybuilders also reported using an additional competition day strategy, e.g., regular diet and the consumption of high GI CHO pre-stage. The consumption of high GI CHO prior to taking to the competition stage was the most popular competition day strategy. On the competitive stage, bodybuilders are required to perform sustained isometric contractions via mandatory poses for five to 20 min. These isometric contractions are likely to have a high glucose demand and the intake of additional CHO pre-stage seems sensible [41]. Moreover, bodybuilders typically “pump up” (increase the volume of blood concentrated in a muscle by exercising and ergo briefly increasing the size of the muscle) 30 to 60 min before competing by performing high repetition resistance training [10,12]. Competitors consume CHO alongside the pump up with belief that it may enhance the muscle pump achieved through cellular swelling [42]. This approach may have some merit amongst competitors with depleted muscle glycogen; however, in competitors practicing CHOL, the additional CHO likely contributes to blood glucose. Some competitors also sodium loaded during the pump up phase, presumably with the same goal in mind. Acute sodium loading is known to increase blood pressure so there may be some rationale for this approach [43]. The persistence of high CHO diets from peak week also reflects the notion that competitors seek to consolidate the fine tuning achieved during peak week as competitors noted the effect CHO had on their muscle size. The bodybuilders’ competition day requirements are unknown, although this higher CHO intake is consistent with recommendations for bodybuilding training (4 to 7 g/kg BW) [44]. Interestingly, some competitors consumed alcohol on competition day. Bodybuilders routinely exclude alcohol from their regular weight loss diet, although it’s use as a competition day diuretic is reported in lay literature [3,11,45]. Bodybuilders may also be using alcohol for psychological reasons. Bodybuilders compete in minimal clothing in front of audiences and judges; it is possible that some competitors consume alcohol to reduce the stress of competition.

Fibre restriction via the removal of fibrous vegetables was also prevalent on competition day, as competitors sought to prevent bloating, and maintain a flat waist. Fibre is noted for its ability to increase water retention, and its restriction is recommended for athletes seeking to make weight [29]. Fibre fermentation by the gut microbiota produces short chain fatty acids, which are known to aid sodium and fluid absorption [46]. Reducing fibre intake may therefore be a viable means of achieving the above-mentioned goal. Finally, some competitors utilised high protein and fat meals on the morning of their competition. Some competitors noted utilising a “Fry up”, a meal consisting of processed meats, eggs, and fried vegetables as a contest day strategy, while grazing on combinations of rice cakes, preserves, peanut butter, and red and white meat was also reported. These additional fats on competition day likely provide bodybuilders with a valuable source of energy. Although bodybuilders should be cautious about food items they may have previously excluded, particularly those high in sodium, as the effect on their appearance may be difficult to predict when other competition day strategies are also employed.

### 4.3. Practical Implications, Safety, and Limitations

Bodybuilders and coaches should consider peaking strategies with caution. They should be mindful that peaking can be stressful, risky, and may negatively affect a competitor’s appearance [6,8]. This manuscript should not be considered a guide to peaking; it merely attempts to describe current practices and the plausible mechanisms of action. The strategies reported in the present investigation may also not reflect the most effective strategies to enhance a competitor’s aesthetics. Indeed, not all competitors surveyed reported employing a peaking strategy and it is possible that other strategies exist and may be more prevalent in different parts of the world. For example, British natural bodybuilders are known to place a greater emphasis on adherence to the World Anti-Doping Agency code than their American counterparts [47]. This difference in what constitutes “natural” or drug-free may result in differences in dieting and peaking strategies across the Atlantic. Additionally, in non-drug tested bodybuilding, competitors’ may make use of pharmaceutical diuretics and anabolic steroids, without medical supervision; further implications of androgenic steroid use are potential water retention [48]. Pharmaceutical diuretics use alongside water and electrolyte manipulation presents an additional danger and professional bodybuilders have died following complications associated with their use [10,12]. The use of diuretics and other performance enhancing compounds amongst non-tested bodybuilders may result in athletes taking a different approach to peak week [14]. Moreover, hyponatraemia has been observed in natural athletes over consuming water without the addition of electrolytes (i.e., plain water), and, in the case of those attempting to make weight via diuresis, deaths have occurred [49,50,51].

The present investigation is not without limitations. Although some athletes provided qualitative data to accompany the quantitative data collection, this data was often provided as short statements rather than detailed accounts. As a result, the exact nature of many of the plans followed could not be quantified. The qualitative data, however, provides useful context on how these plans compare to established strategies [11]. Readers should also be mindful that the data from female competitors reflects athletes competing in different classes (athletic, figure, and bodybuilding). Different physique classes place different expectations on their athletes, which likely influence peaking practices. This fact meant no comparison in peaking methods was made between the male and female cohort. Despite these limitations, the present investigation reported a high prevalence of peaking strategies amongst British natural bodybuilders. Researchers should be mindful of the real-world practices of bodybuilders and seek to understand these strategies. These peaking strategies were ostensibly classic CHOL combined with water manipulation [11,12]. It is possible that experienced bodybuilders can enhance their appearance through peaking, while those considering peaking would be best advised to trial strategies in advance of the competition. Finally, bodybuilders and coaches should be mindful of bodybuilding lore stating that peaking is only likely to be effective if the athlete is suitably conditioned [11].

## 5. Conclusions

Peaking and competition day strategies amongst British natural bodybuilders are common. Carbohydrate and water manipulation were the most frequently employed strategies in the present investigation, while electrolyte manipulation was utilised to lesser extent. Moreover, although a small percentage of competitors opted to follow their regular diet during peak week, many of these competitors still employ a competition day strategy. The efficacy of these strategies to improve contest day performance is unknown, however, the persistence amongst bodybuilders suggests peaking strategies may have some merit. The subjective nature of competitive bodybuilding however, makes this last point difficult to quantify. The fact that peaking is so prevalent amongst bodybuilder may make cautioning peaking unrealistic, and nutritionists and coaches should attempt to understand these strategies to better advice competitors. This is the first manuscript that attempts to document and describe peaking practices utilised by competitive natural bodybuilders. Its findings are likely to be of interest to coaches and athletes involved in bodybuilding. Future work should concentrate on the metabolic requirements of competition day bodybuilding to better prepare athletes for their time on stage. Data observing larger numbers of competitors from each of the different classes would add to the present analysis. Finally, more qualitative research is required to better understand the role peaking plays in bodybuilding culture.

## Figures and Tables

**Table 1:** Characteristics of British competitive natural bodybuilders.

	Males *n*–59		Females *n*–22	
	Mean	±	Mean	±
Age	33.02	12.00	34.74	9.70
Years Training	12.48	9.49	5.29	6.16
Years Competing	3.56	3.14	2.30	1.26
Diet Length (weeks)	22.68	9.45	23.65	6.91
Height (m)	1.77	0.06	1.63	0.05
Diet Start Weight (kg)	88.32	10.14	63.82	6.72
Diet End Weight (kg)	76.57	78.10	55.20	5.22
Total Weight Loss (kg)	11.73	5.55	8.62	3.40
Weight Loss Per Week (kg)	0.63	0.55	0.39	0.16
% Weight Loss	13.00	5.55	13.31	4.48
% Weight Loss Per Week	0.72	0.66	0.60	0.24
End BMI (kg/m^2^)	24.55	1.79	20.63	1.27

Abbreviations: ± standard deviation, m = meters, kg = kilogram, % = percentage, BMI = body mass index.

**Table 2:** Prevalence of peak week strategies amongst British competitive natural bodybuilders.

	Carbohydrate			Water			Sodium					
	Restriction	Loading	Both *	Loading	Restriction	Both *	Restriction	Loading	Both *	Vit. C	Reg. Diet	‘Other’
Males n–59	34	46	28	38	16	12	9	13	4	14	5	5
	57.6%	78.0%	47.4%	64.4%	27.1%	20.3%	15.3%	22.0%	6.8%	23.7%	8.5%	8.5%
Females n–22	18	21	17	15	10	8	2	2	1	5	0	0
	81.8%	95.5%	77.3%	68.2%	45.5%	36.4%	9.1%	9.1%	4.5%	22.7%	0.0%	0.0%
Total n–81	52	67	45	53	26	20	11	15	5	19	5	5
	64.2%	82.7%	55.0%	65.4%	32.1%	25.0%	13.6%	18.5%	6.2%	23.5%	6.2%	6.2%

Results are expressed as total counts and percentages. Abbreviations, Vit C = vitamin C loading is practiced, Reg. Diet = regular competition diet is followed. ‘Other’ examples include, protein and fat loading, and large amounts of dandelion tea consumption. * represents the number and percentage of competitors who employed both restriction and loading. Note the columns above represent the order in which loading and restriction are practiced, i.e., water loading typicaly preceeds water restriction.

**Table 3:** Indicative quotes about peak week strategies from British competitive natural bodybuilders.

Peek Week Strategy	Counts of Qualitative Text	Indicative Quotes
Carbohydrate Restriction	54	“*At the start of peak week I would switch back to low carbs until 3 days out*”, “*I gradually increase my water load the week before contest day and also carb deplete in that week, for around 3 days, depends on my looks and the final 2 days before I carb load, being 2.5 times more than my normal carb intake*”, “*Three day deplete, high fibre and protein*”, “*Deplete 3 days….carbs 100 > 75 > 60 g*”, “ *4 day carb deplete*”, “*3 days, 1/2 carbs every day*”,
Carbohydrate Loading	64	“*Carb loaded 2 days before using high GI* (glycemic index) *carb + rice. Increased water on these days*”, “*4 days out a mix of simple and complex carbs, 1100 g, 600 g, 400 g, 700 g*”, “*three day load, high GI initially followed by low GI 2500 g over 3 days*”, *Load 3 days….carbs 1200, 800, 500 g*”, “*Carb and water load 3 x maintenance level*”, “*Carb up slowly for 3 days using sweet potatoes, rice cakes, jam*”
Water Loading	42	“*I water load on peak week while increasing vitamin C, then drop water back down*”, “*1 day 12 L and then lower at 8 L then 4 L*”, “*10 L for 7 day out*”, “*Water 8 L day, stop consuming 10 p.m. evening before show*”, “*up to 8 L Thurs, 7 L Fri, taper off Saturday*”
Water Restriction	26	“*Cut water 24 h from show just sip*”, “*the day before cut water out*”, “ *Stop water a 3 p.m. day before show–glass of wine night before and sip an wine day of show*”, “*Water reduction from Friday (Sunday competition)”, “Cutting water around 6 p.m. (night before competition)*”, “*Night before show I cut water, sipping with carb meals only*”
Sodium Depleting	10	“*Salt gradually reduced last 3 days below 1 g Na/day*”, “*Stopped salt 3 days before comp.*”, “*No salt the last 3 days*”, “*No salt all during the week*”
Sodium Loading	16	“*Salting meals–pink salt all week*”, “*Increased sodium for 4 days*”, “*salt high till day before then lower water + drop salt*”, “*On contest day I load up with salt (sodium)*”, “*relative to CHO + water*”
Vitamin C Loading	17	“*4 days out 2 g, 3 days out 4 g, 2 days out 6 g, 1 day out 8 g*”, “*Throughout days 2 and 3, vitamin C and water loading over…two days before show vit C increases accordingly*”, “*Increase water ….1:1 ratio of 1000 mg of vit C, then drop water to ½ day before keeping vit C at 5000 mg*”, “*up to 2000 mg daily 4 days pre comp*”
Regular Diet is Followed	5	“*No I believe in sticking to my diet plan right until the end, its never led me wrong, but I would be open to trying other things on show day*”, “*No, we didn’t change much*”, “*No major changes to overall routine*”

**Table 4:** Prevalence of competition day strategies amongst British competitive natural bodybuilders.

	High GI CHO. Pre Stage	Higher CHO.	Water Restriction	Minimal Fibre	Alcohol	High Protein/Fat Grazing	Sodium Loading	‘Other’
Male n–59	40	27	12	11	9	5	9	8
	67.8%	45.8%	20.3%	18.6%	15.3%	8.5%	15.3%	13.6%
Females n–22	16	5	6	5	6	3	2	3
	81.8%	22.7%	27.3%	22.7%	27.3%	13.6%	9.1%	13.6%
Total n–81	59	32	18	16	16	8	11	11
	71.6%	39.5%	22.2%	19.8%	18.5%	9.9%	13.6%	13.6%

‘Other’ strategies include: Water loading, the consumption of B-vitamins, the use of arginine based supplements, CHO restriction, and food restriction. Abbreviations: GI = Glycaemic Index, CHO = carbohydrate.

**Table 5:** Foods consumed by British competitive natural bodybuilders on competition day.

High Glycaemic Index Carbohydrates Pre-Stage	Grapes, Orange Juice, Jaffa cakes, Dark Chocolate, Wine gums, Jelly Babies, Haribo, Skittles, Honey, Jam, Jelly, Syrups, Rice cakes, Dextrose, Glucose
Carbohydrate Sources	White Potatoes, Sweet Potatoes, Buckwheat, Rice, Oats, Rice Cakes, Marmite (Yeast Extract), Baby Food, Salted Crisps, Cookies
Protein and Fat Sources	Almonds, Peanut butter, Cashew butter, Poached Eggs, Steak, Chicken, Turkey, Fry up
Alcohol	Brandy, Whisky, Vodka Red/White Wine

Pre-Stage carbohydrates are consumed in the 30 to 60 min period prior to competitors taking the stage to compete.

**Table 6:** Indicative quotes about competition day strategies amongst British natural bodybuilders.

Strategy	Counts of Qualitative Text	Indicative Quotes
Pre-stage Carb.	55	“*back stage 20 mins before stage–skittles/ sugary sweets, 10 mins before stage pump up*”, “*20 mins before stage–sugar fix*”, “*Follow regular diet, eat sweets before going on stage*”, “*10 mins before stage 20 g dark chocolate” “Haribo while pumping up back stage*”
Higher Carb.	25	“*rice cakes on honey*”, “*I eat 100 g of chocolate on competition day, sweet potatoes, buckwheat and rice cakes*”, “*I eat rice cakes and peanut butter jam every 2 h before judge*”, “*high carb every 2 h*”, “*not really loading but large preserve. mainly sweet potato*”
Water Restriction	11	“*Minimum water on comp day*”, “*Water only to quench thirst*”, “*Sip water only*”, “*Just sipped water as needed” “Nil water*”, “Water cut 6 p.m. day before comp, then sips with food only” *Water depleting?* “minimum on Sunday”, “*I limit my intake to around 500 mL pre-judging thereafter I had a litre for the evening for the show*”
Fibre Restriction	10	“*My fibre intake is really low on contest day to stop bloating*”, “*minimal veg, easily digestible food*”, “*No fibrous veg*”, “*Dropped veggies 24 h pre show*”, “*Minimal fibre” “Removal of green veg and oats*”
Comp. Day Alcohol	15	“*Before going on stage I will have rice cakes, a few sweets and a glass of wine*”, “*Whilst pumping up, I will sugar load my system and may have a sip of whisky*”, “*minimum carbs and a whisky before stage*”, “*Pre stage: red wine + Haribo/Dark Chocolate*”, “*Pre-evening show I have a few sips of red wine*”
High Protein and Fats	8	“*Breakfast–fats + protein (eggs +bacon)*”, “*Small amount of steak + rice cakes throughout the day*”, “*Steak and 2 eggs for breakfast then just graze during the day on rice cakes and honey*”, “*Healthy fats and chicken for all meals on competition day*”, “*Fry up for breakfast (sodium + fat)*”
Sodium Loading	12	“*I had about 1500 mg sodium about an hour before going on stage*”, “*Immediately before stage - salt + grapes*”, “*1 tsp salt prior to stage*”, “Upped salt on all meals”, *use of sodium or salt foods?* “3 g in oats”, “*Salty crisps and dark chocolate 30 to 40 min before the stage*”.
Regular Diet or Other	17	“*Regular diet this time*”, “*follow regular prep, add in extra grapes 20 min before stage*”, “*no plan*”, “*Followed regular diet mainly, add extra fat at breakfast for energy*”, “*foods low to prevent bloating*”, “*Nitrix oxide prior to stage*”

## Supplementary Materials

The following are available online at http://www.mdpi.com/2075-4663/6/4/126/s1, Supplementary Materials S1, Dietary assessment questionnaire used to carry out this research.
